# HOCPCA Exerts Neuroprotection on Retinal Ganglion Cells by Binding to CaMKIIα and Modulating Oxidative Stress and Neuroinflammation in Experimental Glaucoma

**DOI:** 10.1007/s12264-025-01417-0

**Published:** 2025-06-02

**Authors:** Panpan Li, Xin Shi, Hanhan Liu, Yuan Feng, Xiaosha Wang, Marc Herb, Haichao Ji, Stefan Wagner, Johannes Vogt, Verena Prokosch

**Affiliations:** 1 Department of Ophthalmology, Faculty of Medicine and University Hospital of Cologne, University of Cologne, Cologne, 50937 Germany; 2 Key Laboratory of Yunnan Province, Yunnan Eye Institute, Affiliated Hospital of Yunnan University, Yunnan University, Kunming, 650021 China; 3 Department of Orthopedics, The First People’s Hospital of Yunnan Province, Kunming, 650032 China; 4 The Affiliated Hospital of Kunming University of Science and Technology, Kunming, 650032 China; 5 Institute for Medical Microbiology, Immunology and Hygiene, Faculty of Medicine and University Hospital of Cologne, University of Cologne, Cologne, 50935 Germany; 6 Cologne Cluster of Excellence on Cellular Stress Responses in Aging-Associated Diseases (CECAD), Cologne, 50931 Germany; 7 Molecular and Translational Neurosciences, CECAD Cluster of Excellence, CMMC Center of Molecular Medicine Cologne, University of Cologne, Cologne, 50931 Germany

**Keywords:** CaMKII, HOCPCA, RGCs, Glaucoma, Neuroprotection

## Abstract

Neuronal injury in glaucoma persists despite effective intraocular pressure (IOP) control, necessitating neuroprotective strategies for retinal ganglion cells (RGCs). In this study, we investigated the neuroprotective role of the γ-hydroxybutyrate analog HOCPCA in a glaucoma model, focusing on its effects on CaMKII signaling, oxidative stress, and neuroinflammatory responses. Retinal tissue from high IOP animal models was analyzed *via* proteomics. *In vitro* mouse retinal explants were subjected to elevated pressure and oxidative stress, followed by HOCPCA treatment. HOCPCA significantly mitigated the RGC loss induced by oxidative stress and elevated pressure, preserving neuronal function. It restored CaMKIIα and β levels, preserving RGC integrity, while also modulating oxidative stress and neuroinflammatory responses. These findings suggest that HOCPCA, through its interaction with CaMKII, holds promise as a neuroprotective therapy for glaucoma.

**Supplementary Information:**

The online version contains supplementary material available at 10.1007/s12264-025-01417-0.

## Introduction

Glaucoma, a complex neurodegenerative disorder, is principally marked by the progressive loss of retinal ganglion cells (RGCs), leading to significant visual impairment and ultimately blindness. It is projected that by 2040, glaucoma will impact ~111.8 million individuals worldwide [1]. Key risk factors include aging and elevated intraocular pressure (IOP), the latter being the only risk factor currently amenable to modification [2–4]. Despite interventions to reduce IOP, RGC damage often continues, highlighting the irreversible nature of the loss, since RGCs are incapable of regeneration [5–7]. Consequently, the development of neuroprotective strategies is critical [8, 9]. Additional pathological mechanisms contributing to RGC death include neuroinflammation, dysregulated Ca^2+^ homeostasis, alterations in nitric oxide metabolism, and oxidative stress, all of which intensify the progression of retinal neurodegeneration [10].

Recent interest has focused on Ca^2+^/calmodulin-dependent kinase II alpha (CaMKIIα) as a key target for innovative neuroprotective treatments in the central nervous system, including applications in the visual system [11–13]. This kinase is integral to neuronal survival, serving as a crucial regulator of both physiological and pathological Ca^2+^ signaling subsequent to glutamate receptor activation. Altered neuronal activity in inflammatory environments has been attributed primarily to the synergistic interaction between inflammation and the activation of microglial Ca^2+^ channels [14]. CaMKIIα plays a critical role in the regulation of microglial activation. In the CaMKIIα-iCre transgenic mouse model, abnormal activation of microglia leads to severe neuroinflammation and synaptic deficits, particularly in the hippocampus [15]. The N-methyl-D-aspartate (NMDA)-type glutamate receptor induces CaMKII protein phosphorylation and mobilizes intracellular Ca^2+^, thereby activating microglia to exert neurotoxic and pro-inflammatory properties [16]. Compounds such as γ-hydroxybutyrate (GHB) and its structural analog, 3-hydroxycyclopent-1-enecarboxylic acid (HOCPCA), have been identified for their selective targeting [17] and stabilization of the CaMKIIα hub domain [11, 12]. GHB, a derivative of gamma-aminobutyric acid, is noted for its neuroprotective properties across various mammalian models [18–20]. Similarly, HOCPCA selectively binds to high-affinity GHB binding sites and has demonstrated substantial neuroprotective effects in CaMKIIα-activated pathological states, such as ischemic CNS injuries and improvements in cognitive and sensorimotor functions following middle cerebral artery occlusion (MCAO). This is believed to occur through a reduction in the inflammatory response mediated by CaMKIIα [11, 12, 21].

Despite promising data, the specific mechanisms through which these agents act on retinal diseases remain largely undefined. In this study, we aimed to investigate the neuroprotective potential of HOCPCA in the context of glaucoma-related neurodegeneration and to delineate its mechanisms of action. We specifically seek to understand how HOCPCA influences Ca^2+^ signaling and mitigates the neuroinflammatory effects induced by microglia within the retina.

## Methods

### Experimental Animals

In the present investigation, male *C57BL/6J* mice aged 8 and 30 weeks were utilized. The research protocols involving these animals were in strict accordance with the Declaration of Helsinki, complied with the ARVO Statement for the Use of Animals in Ophthalmic and Vision Research, and adhered to the regulations of the German Animal Protection Act. Approval for the protocols was given by the state agency for animal welfare in North Rhine-Westphalia (Landesamt für Natur, Umwelt und Verbraucherschutz Nordrhein-Westfalen, with permission number: 81-02.04.2020. A490), which reviewed and sanctioned the experimental procedures.

### Episcleral Vein Occlusion to Induce Elevated IOP (H-IOP)

In the research, a glaucoma model was established *in vivo* by blocking the three episcleral veins in the right eye of each mouse, as previously noted [22, 23]. The experiment commenced with the sedation of male *C57BL/6J* mice at 8 weeks old, using an intraperitoneal injection containing ketamine (100 mg/kg) and xylazine (10 mg/kg). Oxybuprocain hydrochloride at 4 mg/mL (Novesine® 0.4% Eyedrops, OmniVision GmbH, Puchheim, Germany) was then administered as a single drop to anesthetize the surface of the eye. A conjunctival and Tenon’s capsule incision exposed the episcleral veins, which were cauterized using a thermal cautery instrument (Fine Science Tools GmbH, Heidelberg, Germany). The conjunctiva was subsequently restored to its original position, and ofloxacin ointment was applied for anti-inflammatory purposes. A sham operation identical to the main operation but without episcleral vein damage was also performed. Two weeks following the surgery, the animals were euthanized *via* cervical dislocation.

### Preparation of Retinal Explants

Male *C57BL/6J* mice were euthanized by cervical dislocation; their eyes were excised promptly and placed into ice-cold, sterile phosphate-buffered saline (PBS) in Petri dishes. To expose the retina, the anterior segment of each eye was dissected away, allowing for its separation from the sclera. Following the removal of the vitreous, the retina was carefully detached from the optic cup. The retinal explants were then evenly sectioned into four quadrants, oriented with the ganglion cells facing upward, and positioned on Millipore filters (Millipore, Cork, Ireland).

### HOCPCA Treatment for Ex Vivo Experimental Glaucoma Models

HOCPCA is a cyclic compound with a molecular weight of 159.12 g/mol, containing both hydroxyl and carboxylic acid groups. It exerts its neuroprotective effects through binding to the central domain of CaMKIIα [11, 12, 21]. The sodium salt of HOCPCA, generously provided by Prof. Bente Frølund, was dissolved in the vehicle solution (Dulbecco's modified Eagle's medium/nutrient mixture F-12; DMEM/F12; Gibco BRL, Eggenstein, Germany), enriched with 10 μg/mL porcine insulin, 100 U/mL penicillin, 100 μg/mL streptomycin), and subsequently diluted to various concentrations (1 nmol/L, 10 nmol/L, 100 nmol/L, and 1 μmol/L) for future use. Hereafter, this preparation is referred to as the HOCPCA solution.

**Fig. 1:**
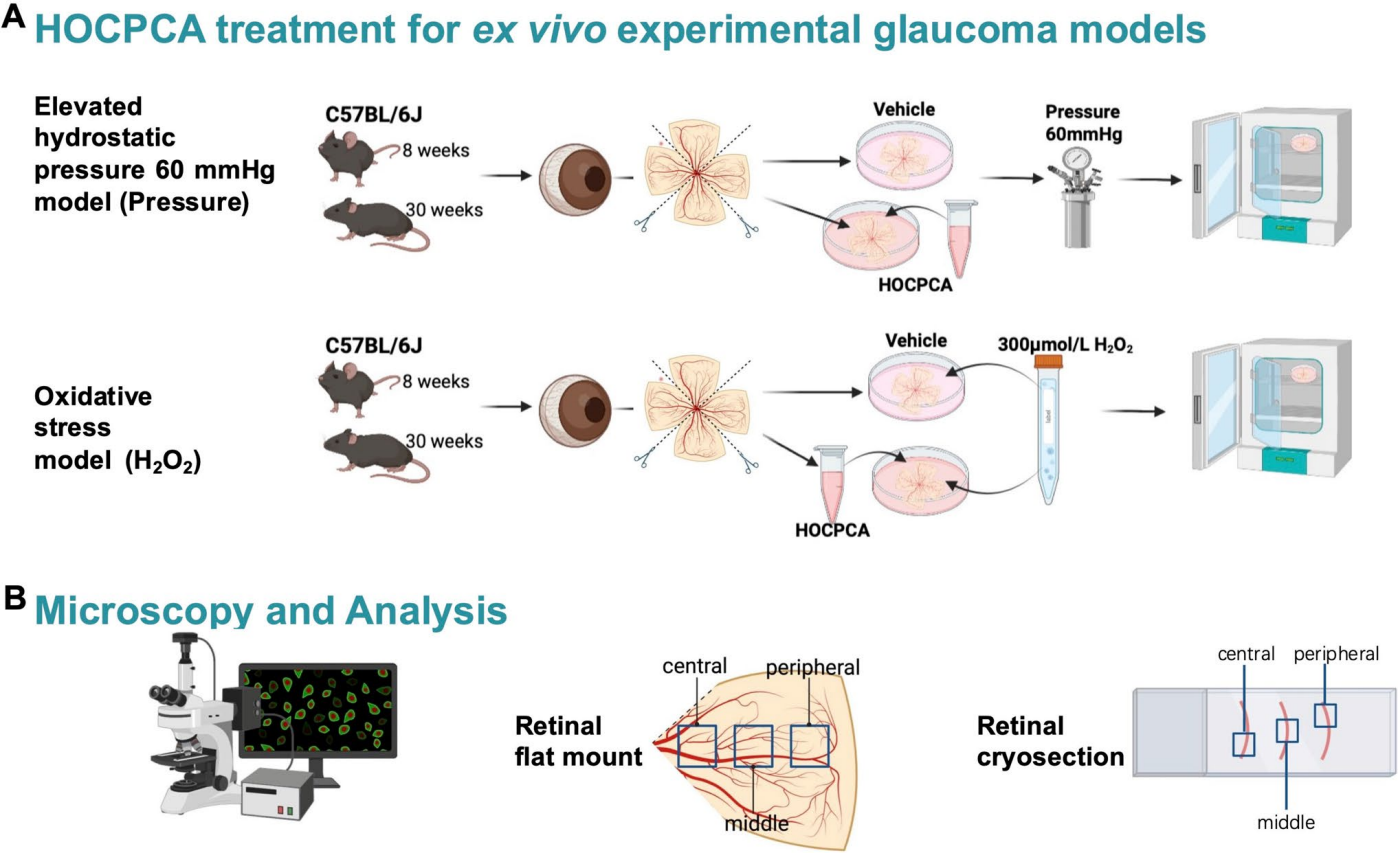
**A** Overview of the *ex vivo* retinal explant Pressure and H₂O₂ model construction, followed by HOCPCA treatment. **B** Overview of imaging and analysis of retinal flat mount and cryosection. The cartoons were created with BioRender.com.

To assess the impact of HOCPCA on RGC survival *ex vivo*, two retinal explant models were investigated: elevated hydrostatic pressure and oxidative stress, alongside a control group. All retinal explants were immediately dissected, prepared, and processed following euthanasia of *C57BL/6J* mice, as per the previously established protocol. Each retina was evenly divided into four parts, resembling petals, as shown in Fig. 1, each quarter retina serving as an independent sample. Fresh retinal explants were immediately used for subsequent *in vitro* experiments. The retinal tissues were then randomly distributed among the control groups and the two experimental models simulating glaucoma to minimize variability. The retinas were immediately placed into lumox Petri dishes 35 (Sarstedt, Nümbrecht, Germany) and subsequently cultured under the respective conditions (Table 1, Fig. 1A).

**Table 1:** Culture Conditions for Retinal Explants in Different Experimental Groups.

Group	Culture Medium	Culture Environment	Duration
Control	Vehicle	5% CO_2_, 37 °C	24 h
Pressure	Vehicle	5% CO_2_, 37 °C+ hydrostatic pressure 60 mmHg	24 h
H_2_O_2_	Vehicle +300 μmol/L H_2_O_2_	5% CO_2_, 37 °C	24 h
Pressure +HOCPCA	HOCPCA solution	5% CO_2_, 37 °C+ hydrostatic pressure 60 mmHg	24 h
H_2_O_2_ +HOCPCA	HOCPCA solution +300 μmol/L H_2_O_2_	5% CO_2_, 37 °C	24 h

*Vehicle:* Dulbecco's modified Eagle's medium/nutrient mixture F-12 (DMEM/F12; Gibco BRL, Eggenstein, Germany) enriched with 10 μg/mL porcine insulin, 100 U/mL penicillin, 100 μg/mL streptomycin*.*

HOCPCA solution*: the sodium salt of HOCPCA dissolved in Vehicle solution.*

The detailed procedures were as follows:

### Control Group

The culture medium in the Petri dishes 35 for the control group was Vehicle solution, which consisted of Dulbecco's modified Eagle's medium/nutrient mixture F-12 (DMEM/F12; Gibco BRL, Eggenstein, Germany) enriched with 10 μg/mL porcine insulin, 100 U/mL penicillin, and 100 μg/mL streptomycin. The retinas were cultured in an incubator with a humidified environment of 5% CO_2_ balanced with air at 37 °C.

### Elevated Hydrostatic Pressure 60 mmHg Model (Pressure)

The retinal explants were cultured in either Vehicle solution or HOCPCA solution. Subsequently, the dishes containing the retinal explants were incubated under a pressure of 60 mmHg for 24 h in a custom-designed pressure culture chamber, simulating the intraocular conditions of abnormally elevated IOP. The chamber, crafted from steel, featured a screw-on lid and a non-directional valve that permitted either the maintenance or adjustment of internal pressure *via* access to 5% CO_2_ balanced with air at 37 °C in the incubator environment. In addition, the pressure within this hyperbaric incubator was continuously monitored by a manometer to ensure stability and accuracy.

### ***Oxidative Stress Model (H***_***2***_***O***_***2***_***)***

For the retinal explants, 300 μmol/L hydrogen peroxide (H_2_O_2_) was incorporated into the Vehicle solution and HOCPCA solution. The samples were then incubated for 24 h in the incubator.

All retinal explants were collected immediately after 24 h in culture under varying experimental conditions, and subsequently processed for further analysis.

### Retinal Tissue Preparation for Cryosection

Retinal explants were initially rinsed in PBS and then fixed in 4% paraformaldehyde (PFA) (Histofix, Roth, Karlsruhe, Germany) for 30 min, followed by overnight immersion in 30% sucrose (sucrose dissolved in PBS). These explants were then flat-mounted on Millipore filters (Millipore, Cork, Ireland). Post-fixation, the retinas were embedded in an optimal cutting temperature compound (Sakura Finetek, Torrance, CA, USA) to prepare them for cryostat sectioning. Using a Leica CM3050S cryostat (Leica Microsystems, Buffalo Grove, IL), the retinal explants were cut vertically into sections 12 μm thick, which were then placed on gelatin-coated slides and kept frozen until they were processed immunohistochemically.

### Immunofluorescence Labeling in Retinal Flat-Mounts and Cryosections

Immunofluorescence staining was carried out using an indirect method. Retinal flat mounts and cryosections were first immersed in a blocking solution containing 5% BSA and 0.3% Triton X-100 in PBS, maintained at room temperature for 60 min. The primary antibodies, listed in Table 2, were mixed with the blocking solution and the sections were then incubated at 4 °C overnight. After incubation, the cryosections were washed three times with PBS for 5 min each to clear any unbound primary antibodies. Subsequently, the corresponding secondary antibody (referenced in Table 2) was prepared in the same blocking solution and applied to the sections for 1 h at room temperature, shielded from light, followed by three 5-min washes. Afterward, the retina flat mount was cleaned, positioned on a glass slide with the RGC layer facing upward, and secured under a glass coverslip using a VectaShield mounting medium containing DAPI (Vector Laboratories, Burlingame, CA).

**Table 2:** Antibodies used for histological analyses.

Antibodies	Manufacturer, Cat. No.	Dilution	Secondary antibody
RBPMS (rabbit)	Novus, NBP2-20112, Lot#130-96	1:300	GαRb A594, 1:1000, Invitrogen
CaMKIIα (6G9) (mouse)	Cell Signaling, #50049	1:200	Gαm A488, 1:1000, Invitrogen Dαm A647, 1:1000, Invitrogen
CaMKIIβ (D-6) (mouse)	Santa Cruz Biotechnology, sc-376828	1:200	Gαm A488, 1:1000, Invitrogen Dαm A647, 1:1000, Invitrogen
CaMKIIγ (rabbit)	Proteintech, #12666-2-AP	1:200	GαRb A594, 1:1000, Invitrogen
CaMKIV (rabbit)	Cell Signaling, #4032	1:200	GαRb A594, 1:1000, Invitrogen
RBPMS (guinea pig)	Millipore Sigma, ABN1376	1:300	GαGp A488, 1:1000, Invitrogen
Iba1 (rabbit)	Fujifilm, #019-19741	1:500	DαRb A647, 1:1000, Invitrogen GαRb A594, 1:1000, Invitrogen
Isolectin GS-IB4	Alexa Fluor 488 conjugate, Invitrogen	1:500	–
Phalloidin-TRITC	Biotechne, #5783	1:500	–
Complement C3	MP Biomedicals, LLC, FITC fluorescein-conjugated goat igg fraction to mouse complement c3, #55500	1:500	–
Factor B (rabbit)	Santa Cruz Biotech, #sc-67141	1:200	GαRb A488, 1:1000, Invitrogen

### Microscopy and Analysis

Images of retinal flat mounts and cryosections were captured using a Zeiss Imager M.2 with an Apotome.2 (Carl Zeiss; Jena, Germany) at a 20× magnification setting. For the retinal cryosections, three consecutive sections from the same sample were imaged, each representing the central, middle, and peripheral regions (Fig. 1B). For the retinal flat mounts, three images were captured for each quarter of the retina, covering the central, middle, and peripheral regions (Fig. 1), to ensure comprehensive analysis. These images were analyzed using ImageJ2 version 2.3.0.

### Mass Spectrometry (MS) Analysis

Protein was extracted from retinal tissue using sodium dodecyl sulfate (SDS), recognized as a highly effective agent for lysing tissues and cells to ensure thorough protein isolation [24]. Following extraction, proteomic analysis was conducted using Data-Independent Acquisition-MS on an LTQ-Orbitrap Elite mass spectrometer known for its high resolution and precision. MS continuum data were collected on an ESI-LTQ Orbitrap XL-MS system (Thermo Scientific, Bremen, Germany), with protein searches against the UniProt database using MaxQuant software, version 1.5.3.30 (Max Planck Gesellschaft, Germany). The inherent variability of label-free quantitative proteomics sometimes referred to as "Birdshot," can lead to occasional absences of protein identification or abundance data in some samples [25]. A false-discovery rate of 0.01 was established for peptide and protein identification, with a minimum peptide length of six amino-acids and at least two unique peptides required. Changes in label-free quantitation intensities were analyzed to determine significant differences in protein expression. Differential expression analysis was applied to the quantitative proteomics data, focusing on H-IOP compared to the control with a threshold of |Student's *t*-test difference| ≥0.5 and a -Log *P*-value >1. Further, profiles of differentially expressed proteins (DEPs) were visualized using volcano plots generated with the R ggplot2 package and hierarchical cluster analysis was conducted using the Manhattan distance metric and the Ward minimum variance method *via* the heatmap package in R. Principal component analysis (PCA) was also applied, utilizing the prcomp function in R, to distill the principal features of the data, serving as an indicator of the overall data condition.

### Gene Ontology (GO) and Kyoto Encyclopedia of Genes and Genomes (KEGG) *Pathway Analysis*

The analysis of differentially-expressed proteins was conducted using GO (http://geneontology.org/), which categorizes findings into three main ontologies: molecular function, cellular components, and biological processes. Concurrently, an examination of biological functions and enrichment pathways was undertaken through the KEGG (http://www.kegg.jp/). Both GO and KEGG analyses utilized R packages that applied hypergeometric distribution models to evaluate the data.

### Quantification of Gene Expression by Quantitative PCR

Quantitative assessment of mRNA levels for Nox enzymes (*Nox2*, *p47*^*phox*^), prooxidant and antioxidant redox enzymes, heme oxygenase 1 (*Ho-1*), cytokines, tumor necrosis factor-alpha (*Tnf-α*), interleukin 1beta (*Il-1β*), and endothelial nitric oxide synthase (*eNos*) was carried out in whole retinal explants as previously detailed [26]. The tissue samples were homogenized using FastPrep equipment (MP Biomedicals, Illkirch, France). RNA was extracted using peqGOLD TriFast™ (PEQLAB), followed by cDNA synthesis with the High-Capacity cDNA Reverse Transcription Kit (Applied Biosystems, Darmstadt, Germany). Quantitative real-time RT-PCR (qPCR) was conducted using the StepOnePlus™ Real-Time PCR System (Applied Biosystems), employing SYBR® Green JumpStart™ Taq ReadyMix™ (Sigma-Aldrich, Munich, Germany) with 20 ng of cDNA. The relative mRNA levels of the genes were quantified by the comparative threshold (CT) method and normalized against the housekeeping gene TATA-binding protein (*Tbp*). Details of the qPCR primer sequences are provided in Table 3.

**Table 3:** Primer sequences used for quantitative PCR analysis.

Gene	Accession number	Forward	Reverse
*Nox2*	NM_007807.2	CCAACTGGGATAACGAGTTCA	GAGAGTTTCAGCCAAGGCTTC
*p47*^*phox*^	NM_001286037.1	AGAGCACGGATGGCACAAAG	CCGCGGGCTGTGGTT
*Ho-1*	NM_010442	GGTGATGCTGACAGAGGAACAC	TAGCAGGCCTCTGACGAAGTG
*Tnf-α*	NM_001278601.1	GCCTCTTCTCATTCCTGCTTG	CTGATGAGAGGGAGGCCATT
*Il-1β*	NM_008361	AAGGAGAACCAAGCAACGACAAAA	TGGGGAACTCTGCAGACTCAAACT
*eNos*	NM_008713	CCTTCCGCTACCAGCCAGA	CAGAGATCTTCACTGCATTGGCTA
*Tbp*	NM_013684	CTTCGTGCAAGAAATGCTGAAT	CAGTTGTCCGTGGCTCTCTTATT

### Primary Neuronal Cell Culture

Dissection of hippocampi and the subsequent culture of isolated hippocampal neurons from day 17 mouse embryos was performed essentially as previously reported [27]. Briefly: Hippocampi were dissected under a stereo microscope, trypsin-digested, and mechanically isolated with fire-polished Pasteur pipettes of decreasing diameter. Cells were seeded in plating medium in 6-well plates at a density of ~80000 cells/cm^2^. Three hours after plating, cells were washed and cultured in a neurobasal medium with a B27 supplement.

### Western Blotting

Hippocampal neurons at 16 days *in vitro* in 6-well plates were washed with PBS and lysed in 100 µl RIPA buffer per well. The retinas were washed twice with cold PBS and incubated in 50 µL RIPA buffer on ice. The protein concentration of each retinal lysate was measured using the BCA Protein Assay Kit (Pierce, Rockford, IL, USA), following the manufacturer’s instructions. In subsequent steps, 80 µg of protein from each lysate was used. The samples were denatured in 5× SDS sample buffer and 10 µL loaded onto 10% SDS acrylamide gels. Following protein transfer to nitrocellulose membranes and antibody incubation (refer to Table 4 for antibody details), target proteins were detected either by fluorescence or chemiluminescence using a Fusion FX (Vilber) detector.

**Table 4:** Antibodies used for Western blotting.

Antibodies	Dilution	Manufacturer, Cat. No.
CaMKIIα (6G9)	1:1000	Cell Signaling, #50049
CaMKIIβ (D-6)	1:1000	Santa Cruz Biotechnology, sc-376828
CaMKIIγ	1:1000	Proteintech, #12666-2-AP
CaMKIV	1:1000	Cell Signaling, #4032
β-actin	1:1000	Abcam, ab6276

### Primary Microglia Culture and Stimulation

As previously described [28], primary microglia were isolated from neonatal C57BL/6J mouse pups (P0). The cell suspension was seeded onto poly-D-lysine-coated culture plates (1 mg/mL stock solution in PBS, diluted to 50 ng/mL for use, #A003E; Nunc), using PM medium (Neurobasal-A, 10% horse serum, B27 supplement, 100 U/mL penicillin, 100 µg/mL streptomycin, and 2 mM glutamine). Third-generation primary microglia were used in the experiments. On day 3 in culture (DIV3), cells were stimulated with 100 ng/mL lipopolysaccharide (LPS; InvivoGen, TLRL-EKLPS) and treated with HOCPCA at concentrations of 100 nmol/L or 1 µmol/L for 24 h. For immunostaining, primary microglia grown on coverslips were processed, washed with PBS, and fixed with 4% PFA for 30 min. The immunostaining protocol for primary microglia was identical to that used for retinal flat-mount staining, and the antibodies used are listed in Table 2. DIV3 primary microglia cultured in 6-well plates were washed with PBS and lysed in 100 µL of RIPA buffer per well for Western blot analysis.

### Enzyme-Linked Immunosorbent Assay (ELISA)

Cytokine concentrations in retinal lysates and the culture supernatants from microglial cells were quantified using ELISA assays. The tissue samples were sonicated in PBS, which was enhanced with protease and phosphatase inhibitors (Complete Protease Inhibitor Cocktail, Roche). The ELISA kits used for measuring IL-1β/IL-1F2 (DY401), TNF-α (DY410), and IL-6 (DY406) were from R&D Systems under the Quantikine® brand.

### Quantification of Cytosolic ROS

The retinas were homogenized in a lysis buffer following the previously detailed method [29]. After centrifugation at 1500 g for 3 min, the supernatant was incubated with 20 μmol/L of the fluorescent probe 6-carboxy-2,7-dichlorodihydrofluorescein diacetate, di(acetoxymethyl ester) (DCF), for 30 min at 37 °C. Fluorescence was assessed using a Tecan infinite 200Pro plate reader, with excitation and emission settings at 485 nm and 535 nm, respectively.

### ***Quantification of Superoxide (O***_***2***_^***-***^***)***

Superoxide levels were determined in unfixed, frozen retinal sections stained with the fluorescent dye dihydroethidium (DHE) [22, 30–32]. Sections were thawed and then incubated with 1 μmol/L DHE for 30 min at 37 °C. Following incubation, the sections were mounted in VectaShield mounting medium (Vector Laboratories, Burlingame, CA) and coverslipped. Images of the stained retinal cross-sections were captured using a Leica SP8 confocal laser scanning microscope (Leica, Wetzlar, Germany). The intensity of staining across different retinal layers was quantified using ImageJ software (NIH, http://rsb.info.nih.gov/ij/), consistent with previous descriptions.

### Statistical Analysis

Data are presented as the mean ± standard error of the mean (SEM), with 'n' indicating the number of samples per group. Statistics were analyzed and graphs were created using GraphPad Prism version 9.0 (GraphPad, San Diego, CA). Specific statistical methods are described in the figure legends. A *P*-value <0.05 was considered to indicate a statistically significant difference.

## Results

### Proteomic Crosstalk of Neuroinflammation and Oxidative Stress with CaMKIIα in H-IOP Retinas In Vivo

**Fig. 2:**
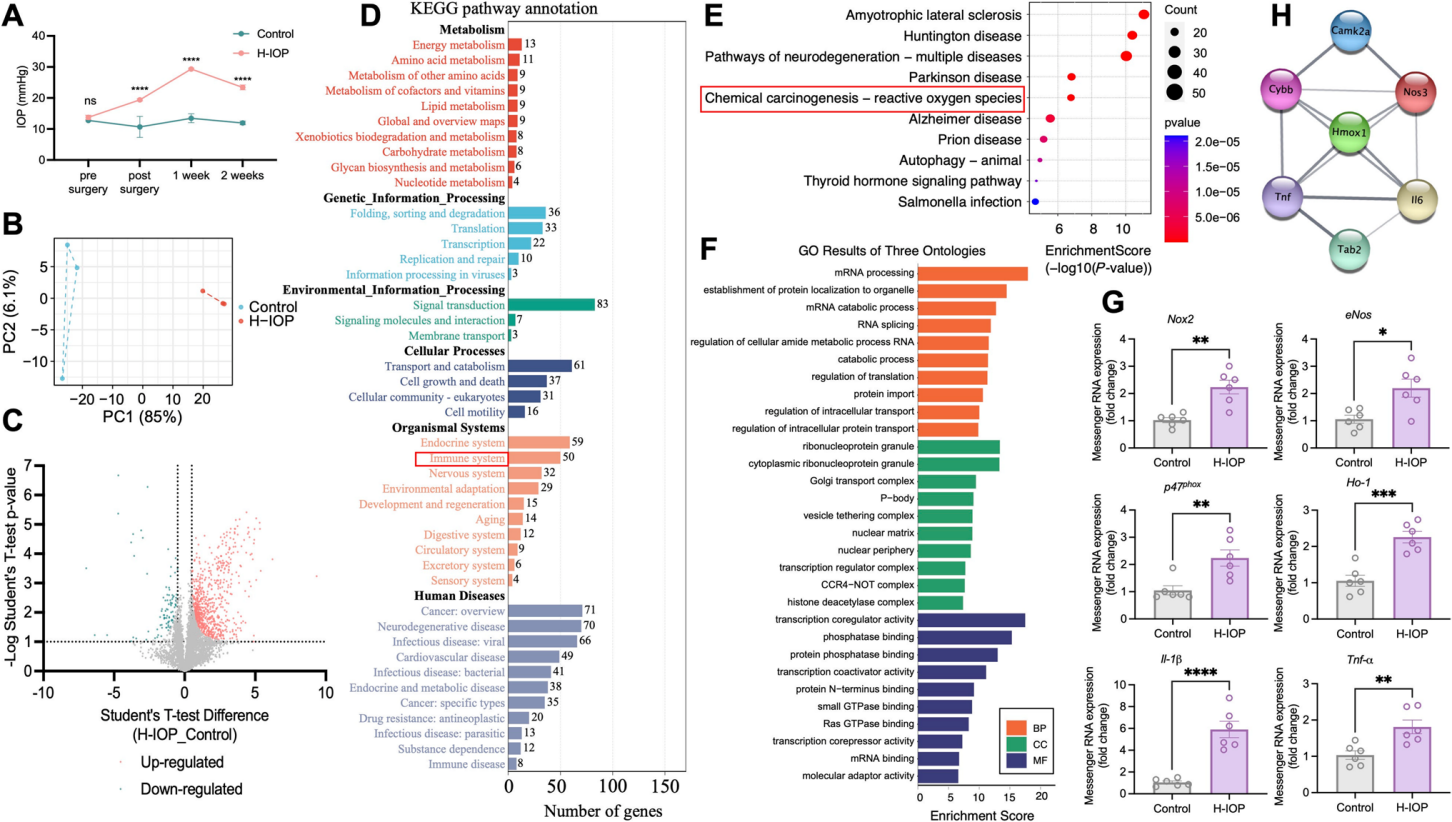
**A** IOP elevation in H-IOP eyes; mean ± SEM (*n =* 3/group, two-way ANOVA with Šídák's multiple comparisons test). **B** Principal component analysis showing protein clustering in different samples. **C** Volcano plot of differentially-expressed proteins between H-IOP and control. **D** KEGG level 2 pathways for 855 differentially-expressed proteins. **E** Top 10 enriched KEGG pathways (symbol size = number of genes; color = *P*-value). **F** Top 10 enriched gene ontology (GO) terms for the parental genes of the differentially-expressed immune genes. Enriched GO = vertical axis, number of annotated differentially expressed genes associated with each GO term = horizontal axis. **G** Messenger RNA expression of retinal oxidative homeostasis markers (*Nox2*, *p47 *^*phox*^*, eNos*, and *Ho-1*) and inflammatory markers (*Il-1β* and *Tnf-α*) in Control and H-IOP retinas. Data are presented as fold-change in retinas after H-IOP *versus* Control (*n =* 6; mean ± SEM; unpaired *t*-test, *P <0.05, **P <0.01, ***P <0.001, ****P <0.0001). **H** PPI network of CaMKIIα with retinal oxidative homeostasis and inflammatory markers (from string-db.org, medium confidence = 0.46).

After episcleral vein occlusion *in vivo* for two weeks, a significant elevation in average IOP was recorded, with the H-IOP eyes reaching 23.40 ± 0.46 mmHg, well above the physiological range found in control eyes (11.93 ± 0.33 mmHg, *P <*0.0001) (Fig. 2A). Proteomic changes were mapped in the H-IOP and control groups (*n* = 3/group). PCA revealed distinct proteomic profiles between H-IOP and control retinas, highlighting differentially-expressed proteins (DEPs) that distinguished the two groups (Fig. 2B). A comparison of proteomic data identified 761 proteins that were significantly more abundant and 94 proteins that were significantly less abundant in the H-IOP group than in controls (Fig. 2C).

KEGG enrichment and secondary pathway analyses of the 855 DEPs (Fig. 2D, E) indicated significant enrichment in immune system pathways. Both KEGG (Fig. 2E) and GO analyses (Fig. 2F) associated the DEPs with oxidative damage responses.

qPCR revealed changes in retinal oxidative homeostasis markers (*Nox2*, *p47*^*phox*^, *eNos*, and *Ho-1*) and inflammatory markers (*Il-1β* and *Tnf-α*). As illustrated in Fig. 2G, the H-IOP group showed significant upregulation of *Nox2*, *p47*^*phox*^, *eNos*, *Ho-1*, *Il-1β*, and *Tnf-α* compared to controls, with *Il-1β* levels being nearly six times higher. Protein-protein interaction (PPI) network analysis using string-db.org (Fig. 2H) highlighted the involvement of CaMKIIα in retinal oxidative homeostasis and inflammatory responses, suggesting that these mechanisms play central roles in the retinal damage induced by H-IOP. CaMKIIα emerges as a promising target for modulating inflammation and oxidative stress interactions.

### HOCPCA Has Neuroprotective Effects Against Elevated Hydrostatic Pressure-induced Retinal Ganglion Cell Damage

Based on our extensive previous *ex vivo* experimental data, the elevated hydrostatic pressure 60 mmHg model reliably replicates the retinal damage in *in vivo* models of H-IOP [33–36]. This model is well-established and highly controllable, simulating high intraocular pressure conditions by applying precise hydrostatic pressure. *In vivo* models can be influenced by complex systemic factors, potentially masking the direct effects of drugs on the retina. Therefore, using an *in vitro* retinal explant model allows for the measurement of drug effects under more controlled experimental conditions, including cellular and molecular changes.

**Fig. 3:**
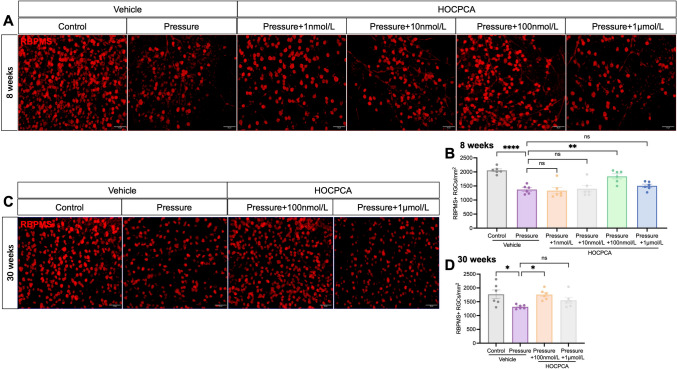
**A** Representative images of immunolabeling with RBPMS (red) in retinal flat-mounts after Pressure application and treatment with HOCPCA for 24 h in 8-week-old retinas. Scale bar, 50 μm. **B** Quantification of RBPMS-positive RGCs in retinas treated with different concentrations of HOCPCA after Pressure in 8-week-old retinas. **C** Representative images of immunolabeling with RBPMS (red) in retinal flat-mounts after Pressure application and treatment with HOCPCA for 24 h in 30-week-old retinas. Scale bar, 50 μm. **D** Quantification of RBPMS-positive RGCs in 30-week-old retinas treated with different concentrations of HOCPCA after pressure. Data are shown as the mean ± SEM (*n =* 6 per group, *P <0.05, **P <0.01, ***P <0.001, ****P <0.0001, one-way ANOVA with Tukey's test).

The retinas from 8-week-old and 30-week-old mice were incubated under Pressure conditions to evaluate its neuroprotective effects on RGCs in various concentrations of HOCPCA. The results demonstrated a significant reduction in the density of RNA-binding protein with multiple splicing-positive (RBPMS+) RGCs in the Pressure group (60 mmHg, 24 h) for both 8-week-old and 30-week-old retinas compared to the control group at baseline pressure (0 mmHg, 24 h) (Fig. 3).

Interestingly, a significant neuroprotective effect was found at a concentration of 100 nmol/L in 8-week-old retinas subjected to elevated pressure (Fig. 3A, B). Low concentrations of HOCPCA (1 nmol/L and 10 nmol/L) did not prevent RGC loss, but at 100 nmol/L, the density of RBPMS+ RGCs (1840 ± 89/mm^2^) was significantly higher than that in the Pressure group (1372 ± 62/mm^2^, *n* = 6, *P <*0.0064). However, this neuroprotective effect was not found at a higher concentration of HOCPCA (1 μmol/L).

Similarly, a protective effect was recorded in 30-week-old retinas, where treatment with 100 nmol/L HOCPCA rescued 34% of the RGCs lost due to elevated pressure (Fig. 3C, D). However, RGC density in retinas treated with 1 μmol/L HOCPCA was not significantly different from that in the Pressure group.

### HOCPCA Protects RGCs Against H_2_O_2_-Induced Damage

**Fig. 4:**
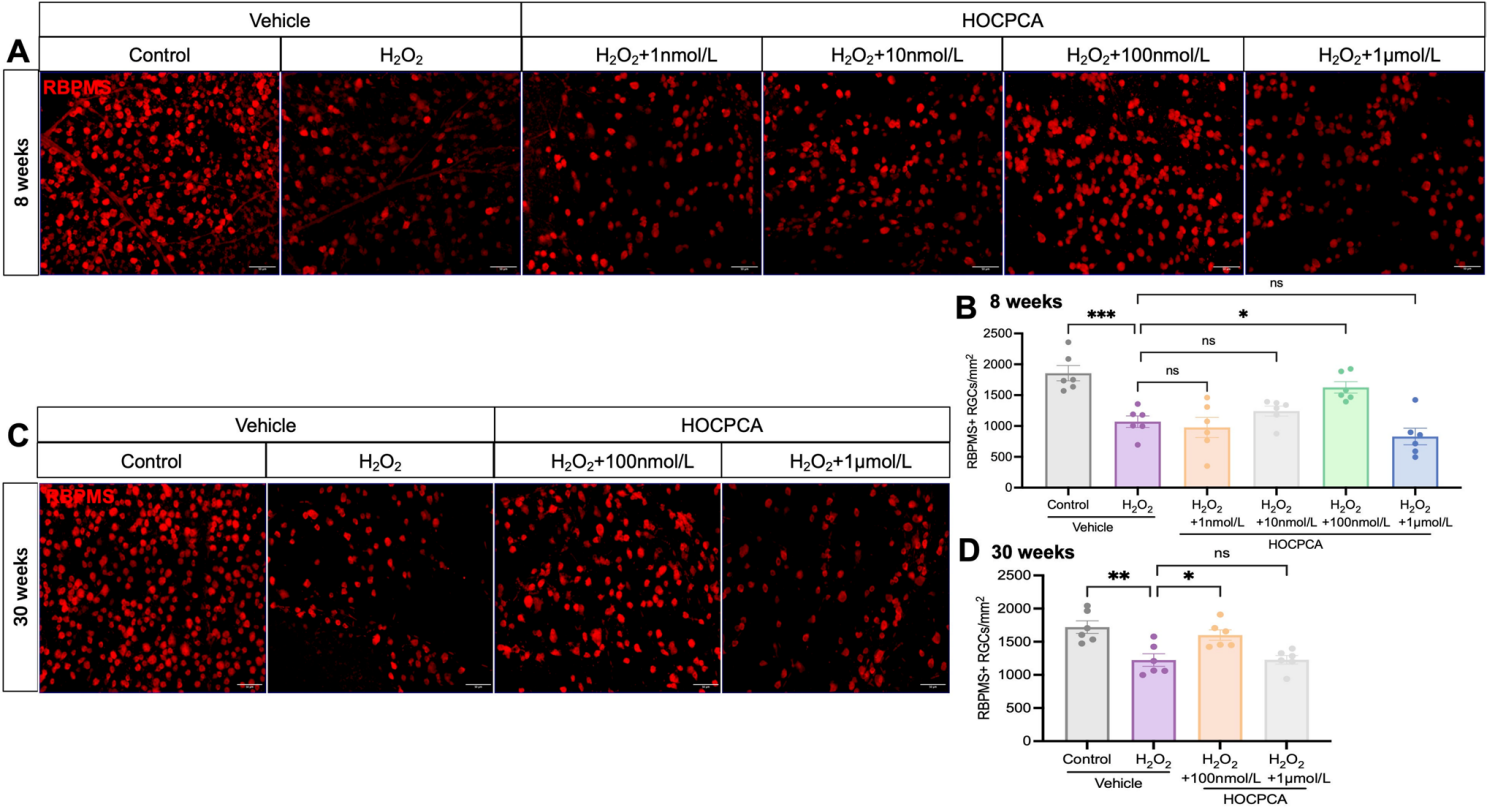
**A** Representative images of RBPMS-positive (red) RGCs in retinal flat-mounts after H₂O₂ exposure and 24-h treatment with HOCPCA in 8-week-old retinas. Scale bar, 50 μm. **B** Quantification of RBPMS-positive RGCs in retinas treated with different HOCPCA concentrations following H₂O₂ exposure in 8-week-old retinas. **C** Representative images of RBPMS-positive (red) RGCs in retinal flat-mounts after H₂O₂ exposure and 24-h treatment with HOCPCA in 30-week-old retinas. Scale bar, 50 μm. **D** Quantification of RBPMS-positive RGCs in 30-week-old retinas treated with varying HOCPCA concentrations after H₂O₂ exposure. Data are shown as the mean ± SEM (*n =* 6 per group, *P <0.05, **P <0.01, ***P <0.001, ****P <0.0001, one-way ANOVA with Tukey's test).

In addition to elevated IOP, oxidative stress is a crucial factor in RGC degeneration. To simulate oxidative stress, we used 300 μmol/L H_2_O_2_, which resulted in a 42% reduction in RBPMS+ RGCs in the 8-week-old retinas and a 29% reduction in the 30-week-old retinas *versus* controls (Fig. 4).

We then explored the neuroprotective potential of HOCPCA against H_2_O_2_-induced oxidative injury. The density of RBPMS+ RGCs significantly increased following treatment with 100 nmol/L HOCPCA compared to H_2_O_2_-injured retinas (Fig. 4). However, at a higher concentration (1 μmol/L), HOCPCA did not enhance RGC survival in either 8-week-old or 30-week-old retinas. Similarly, lower concentrations of HOCPCA (1 nmol/L and 10 nmol/L) were insufficient to rescue RGCs from oxidative stress.

In conclusion, HOCPCA has neuroprotective effects on RGCs in retinas exposed to high pressure and oxidative stress *in vitro*, with significant protection at the specific concentration of 100 nmol/L, while higher and lower concentrations did not have the same efficacy.

### HOCPCA Exerts RGC Neuroprotection by Acting at the CaMKIIα Binding Site

HOCPCA is reported to have significant selectivity and affinity exclusively for the α isoform of CaMKII [12, 37, 38]. To investigate whether HOCPCA exerts its neuroprotective effects through direct interaction with CaMKIIα in RGCs, we analyzed the expression and localization of CaMK family proteins in retinal tissue.

**Fig. 5:**
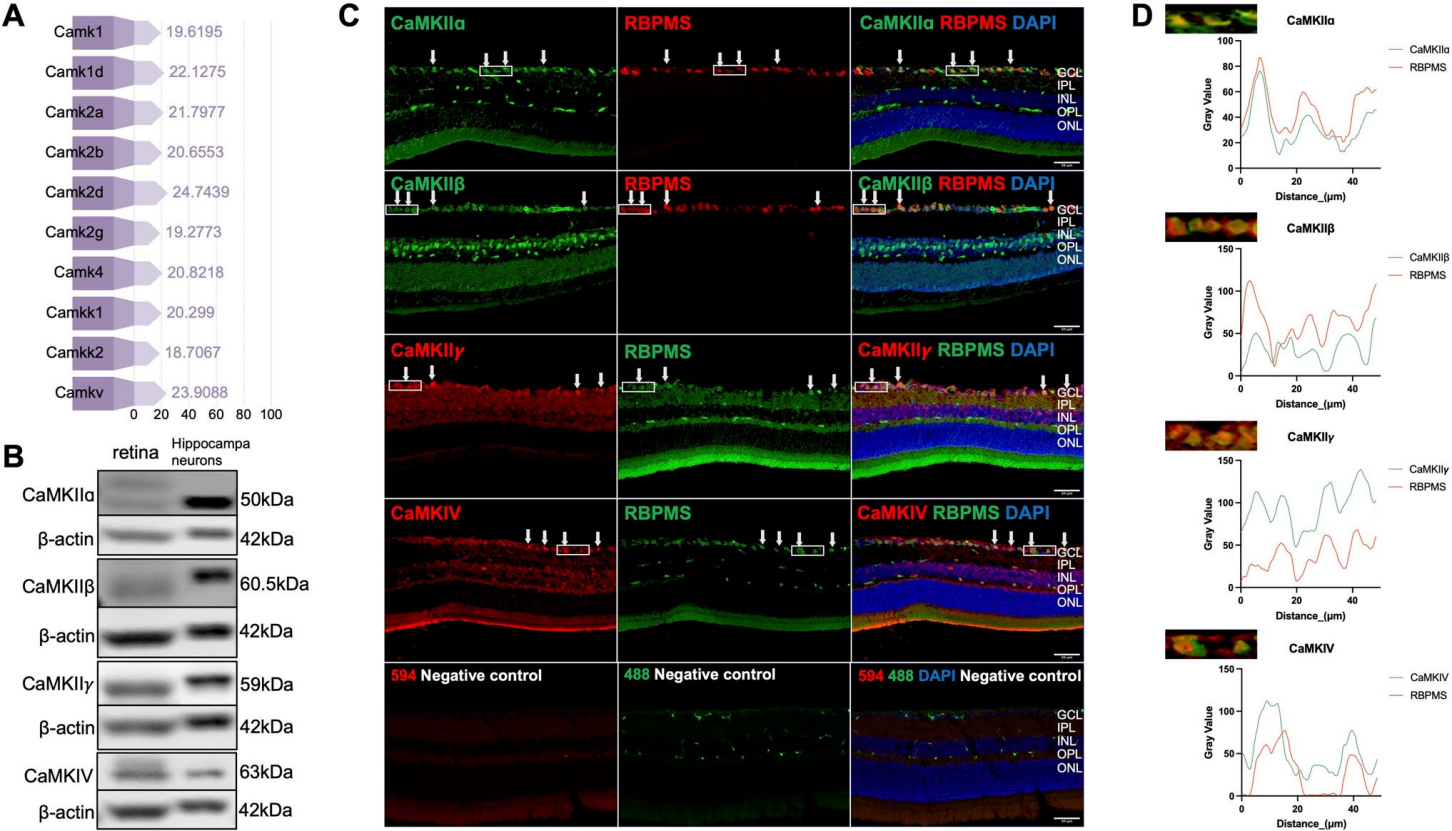
**A** Protein expression profiles in adult mice under normal conditions reveal the presence of 10 CaMK proteins. These include CaMK1, CaMK1d, the four subunits of CaMK2 (α, β, δ, γ), CaMK4, CaMKK1&2, and CaMKV. **B** Western blots of the CaMKIIα, β, and γ isoforms, and CaMKIV in retinal extracts and hippocampal neuron lysates. **C** Representative images of co-labeling for CaMKIIα, β, and γ isoforms, CaMKIV, and RBPMS in retinal cryosections. White arrows, CaMK co-localized with RBPMS; scale bar, 50 μm. **D** The original images shown in the rectangular panel from (**C**) are overlaid with illustrations and intensity profiles measured along vectors across two fluorescent structures, confirming the colocalization of CaMK and RBPMS.

A comprehensive proteomic assessment of retinal tissue under normal physiological conditions in adult mice (Fig. 5A) identified 10 CaMK family proteins: CaMK1, CaMK1d, all four subunits of CaMKII (α, β, δ, and γ), CaMKIV, CaMKK1, CaMKK2, and CaMKV. Western blot analysis using hippocampal neuron lysates as a positive control confirmed the expression of CaMKIIα, β, and γ, as well as CaMKIV, in mouse retinal tissue (Fig. 5B).

Immunofluorescence staining demonstrated that all four CaMK proteins were present in the ganglion cell layer (GCL) and co-localized with RBPMS+ RGCs (Fig. 5C, D). In addition, CaMK expression was detected in the inner nuclear layer (INL), with CaMKIIβ exhibiting a characteristic wedge-shaped pattern.

**Fig. 6:**
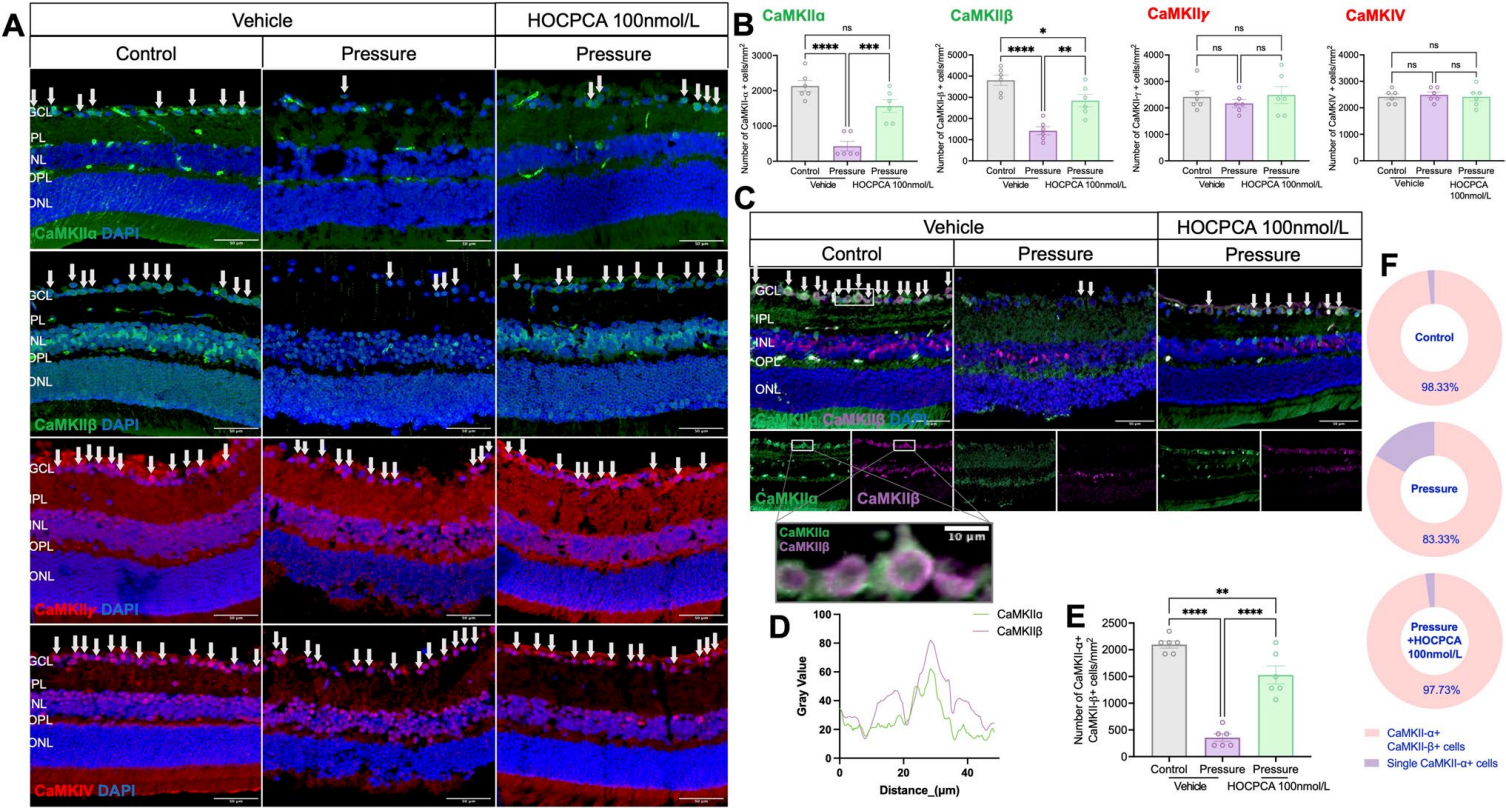
**A** Representative images of immunofluorescence labeling of CaMKIIα, β, and γ isoforms, and CaMKIV in retinal cryosections after Pressure exposure and HOCPCA treatment. White arrows, CaMK-positive cells; scale bar, 50 μm. **B** Quantification of CaMKIIα+, CaMKIIβ+, CaMKIIγ+, and CaMKIV+ cells in the GCL of retinas treated with HOCPCA after Pressure. **C** Representative co-labeling of CaMKIIα and CaMKIIβ isoforms in retinal cryosections after Pressure and HOCPCA treatment. White arrows, CaMKIIα+CaMKIIβ+ cells; scale bar, 50 μm. **D** The original images shown in the rectangular panel from (**C**) are overlaid with illustrations and intensity profiles measured along vectors across two fluorescent structures, confirming the colocalization of CaMKIIα and β. Scale bar, 10 μm. **E** Quantification of CaMKIIα+ CaMKIIβ+ cells in the GCL after HOCPCA treatment under pressure. **F** Composition of CaMKIIα+ cells (CaMKIIα+ CaMKIIβ+ cells and single CaMKIIα+ cells) in the GCL. Data are presented as the mean ± SEM (*n =* 6 per group, *P <0.05, **P <0.01, ***P <0.001, ****P <0.0001, one-way ANOVA with Tukey's multiple comparisons test).

To determine the effects of HOCPCA on CaMK expression under high hydrostatic pressure, we applied immunolabeling to retinal cryosections (Fig. 6A). Quantification revealed a significant reduction in CaMKIIα+ and CaMKIIβ+ RGCs following high pressure *versus* controls (Fig. 6B). Notably, HOCPCA treatment restored CaMKIIα+ cell numbers by 58%, strongly suggesting a protective effect on RGCs mediated through CaMKIIα activation. In addition, the characteristic wedge-shaped expression pattern of CaMKIIβ in the INL was preserved following HOCPCA treatment, despite being significantly reduced under high pressure. In contrast, CaMKIIγ and CaMKIV expression remained unchanged under all conditions (Fig. 6B).

Notably, HOCPCA has been reported to exhibit a 100-fold selectivity for the CaMKIIα binding site [12, 37, 38]. Given this specificity, the preservation of CaMKIIβ+ cells is likely a secondary effect mediated by CaMKIIα+ RGC protection. To further investigate this relationship, we applied double staining for CaMKIIα and CaMKIIβ. In control conditions, 98.33% of CaMKIIα+ RGCs also co-expressed CaMKIIβ. Under high pressure, the proportion of CaMKIIα+CaMKIIβ+ double-positive cells decreased to 83.33% but was fully restored to 97.73% after HOCPCA treatment, similar to control levels (Fig. 6C–F).

These findings indicate that HOCPCA primarily exerts direct neuroprotection on RGCs through its selective binding to CaMKIIα, while the preservation of CaMKIIβ+ cells is likely a consequence of CaMKIIα+ cell survival, indirectly supporting neighboring neurons in the GCL.

### HOCPCA Suppresses Oxidative Stress and Microglia-Induced Neuroinflammation

Previous research has shown that HOCPCA treatment reduces inflammation *via* CaMKII-mediated signaling in the cortex following distal MCAO [21]. To clarify the mechanisms by which HOCPCA provides neuroprotection in the retina under high pressure, we assessed the expression levels of Iba1 (a microglia/macrophage activation marker) and key pro-inflammatory cytokines, including IL-6, IL-1β, and TNF, in the retina.

**Fig. 7:**
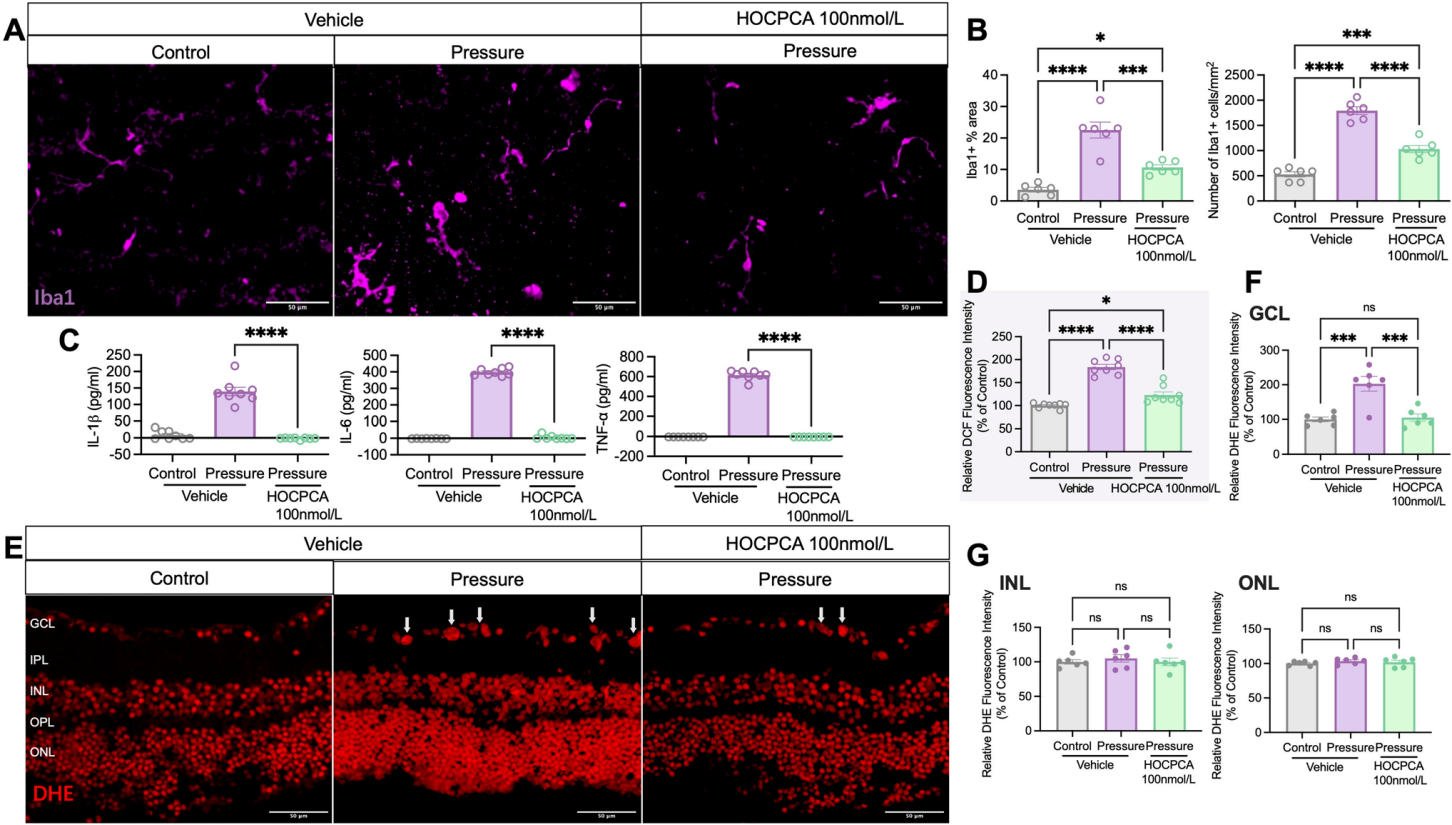
**A** Representative images of Iba1 immunolabeling in retinal flat mounts after Pressure and HOCPCA treatment. Scale bars, 50 μm. **B** The Iba1-positive area and Iba1+ cell density in retinas treated with HOCPCA following Pressure (*n* = 6 per group). **C** ELISA analysis of retinal IL-6, IL-1β, and TNF protein levels after HOCPCA treatment (*n* = 8 per group). Data are shown as the mean ± SEM (*P <0.05, **P <0.01, ***P <0.001, ****P <0.0001, unpaired t-test). **D** Measurement of retinal ROS using the DCF diacetate probe. Data are presented as the percent fluorescence intensity of the groups *versus* the Control (*n =* 8 per group). **E** Representative DHE staining in retinal cross-sections from Control, Pressure, and HOCPCA-treated groups. Scale bars, 50 μm. **F** DHE fluorescence intensity analysis in the GCL. **G** DHE fluorescence intensity analysis in the INL and ONL. Data are normalized to Control values (*n =* 6 per group). Data are shown as the mean ± SEM (*P <0.05, **P <0.01, ***P <0.001, ****P <0.0001, one-way ANOVA with Tukey's test).

Our results demonstrated that HOCPCA significantly inhibited high-pressure-induced activation of Iba1-positive cells in the GCL, markedly reducing both the Iba1-positive area and the density of Iba1+ cells (Fig. 7A, B). Since microglial activation is associated with the release of pro-inflammatory cytokines [39], we quantified IL-6, IL-1β, and TNF levels using ELISA. HOCPCA treatment significantly reduced the elevated levels of these cytokines in pressure-induced retinas (Fig. 7C).

Oxidative stress plays a crucial role in retinal injury under high hydrostatic pressure. To assess HOCPCA’s antioxidant effects, we quantified cytosolic ROS in the retina using the fluorescent ROS probe DCF. Elevated pressure significantly increased ROS production, which was notably suppressed by HOCPCA treatment, although levels did not fully return to control conditions (Fig. 7D). To determine the specific retinal layers affected, we applied DHE staining to assess O₂⁻ production in cryosections (Fig. 7E). The most pronounced oxidative stress occurred in the GCL under hydrostatic pressure, exhibiting a threefold increase, which was significantly reduced by HOCPCA (Fig. 7F), while no significant changes were found in the INL and ONL (Fig. 7G).

**Fig. 8:**
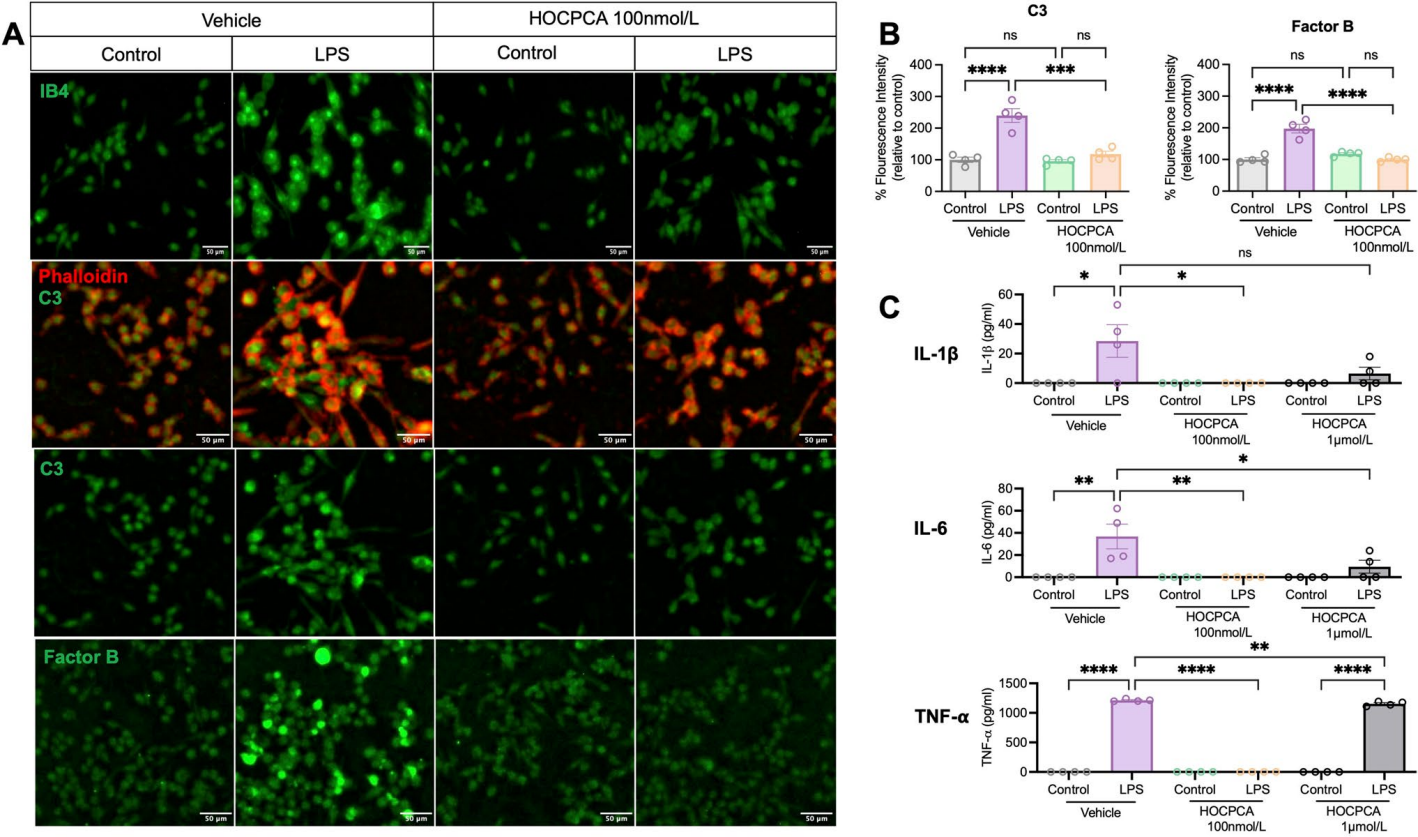
**A** Representative images of microglial morphology labeled with IB4, Phalloidin, complement components C3, and Factor B in primary microglial cultures treated with vehicle or HOCPCA (100 nmol/L), following LPS stimulation. Scale bars, 50 μm. **B** The fluorescence intensity for complement components C3 and Factor B in microglia after LPS stimulation and HOCPCA treatment (*n* = 6 per group). Data are shown as the mean ± SEM (*P <0.05, **P <0.01, ***P <0.001, ****P <0.0001, one-way ANOVA with Tukey's test). **C** ELISA analysis of IL-1β, IL-6, and TNF-α levels in culture supernatants from microglial cells treated with HOCPCA (100 nmol/L and 1 μmol/L) and LPS (*n* = 4 per group). Data are presented as the mean ± SEM (*P <0.05, **P <0.01, ***P <0.001, ****P <0.0001, two-way ANOVA with Tukey's multiple comparisons test).

To investigate the direct effects of HOCPCA on microglial activation, we used a classic primary microglial activation model induced by LPS. The results showed that treatment with 100 nmol/L HOCPCA significantly attenuated LPS-induced microglial activation, as evidenced by morphological changes in cells labeled with IB4 and Phalloidin (Fig. 8A). In the LPS-stimulated group, microglia exhibited pronounced morphological alterations, including increased cell body size and extension of filopodia, whereas HOCPCA treatment effectively reversed these changes, indicating its inhibitory effect on microglial activation.

In addition to the morphological changes, quantification of fluorescence intensity further revealed that HOCPCA at 100 nmol/L significantly reduced the expression of complement components C3 and Factor B in LPS-stimulated microglia (Fig. 8B), highlighting HOCPCA's important role in suppressing complement-mediated microglial responses. Moreover, ELISA analysis showed that LPS stimulation led to substantial secretion of pro-inflammatory cytokines, including IL-1β, IL-6, and TNF-α. Notably, following treatment with 100 nmol/L HOCPCA, levels of IL-1β and IL-6 were significantly decreased (*P <*0.05 and *P <*0.01, respectively), and TNF-α production was drastically reduced (*P <*0.0001) (Fig. 8C). It is worth noting that 1 µmol/L HOCPCA did not effectively inhibit the release of these cytokines, indicating that HOCPCA's anti-inflammatory effects occur only at specific concentrations, consistent with previous findings in RGC quantification.

In summary, these findings confirm that HOCPCA reduces oxidative stress and modulates microglial activity, contributing to a neuroprotective environment in the retina.

## Discussion

**Fig. 9:**
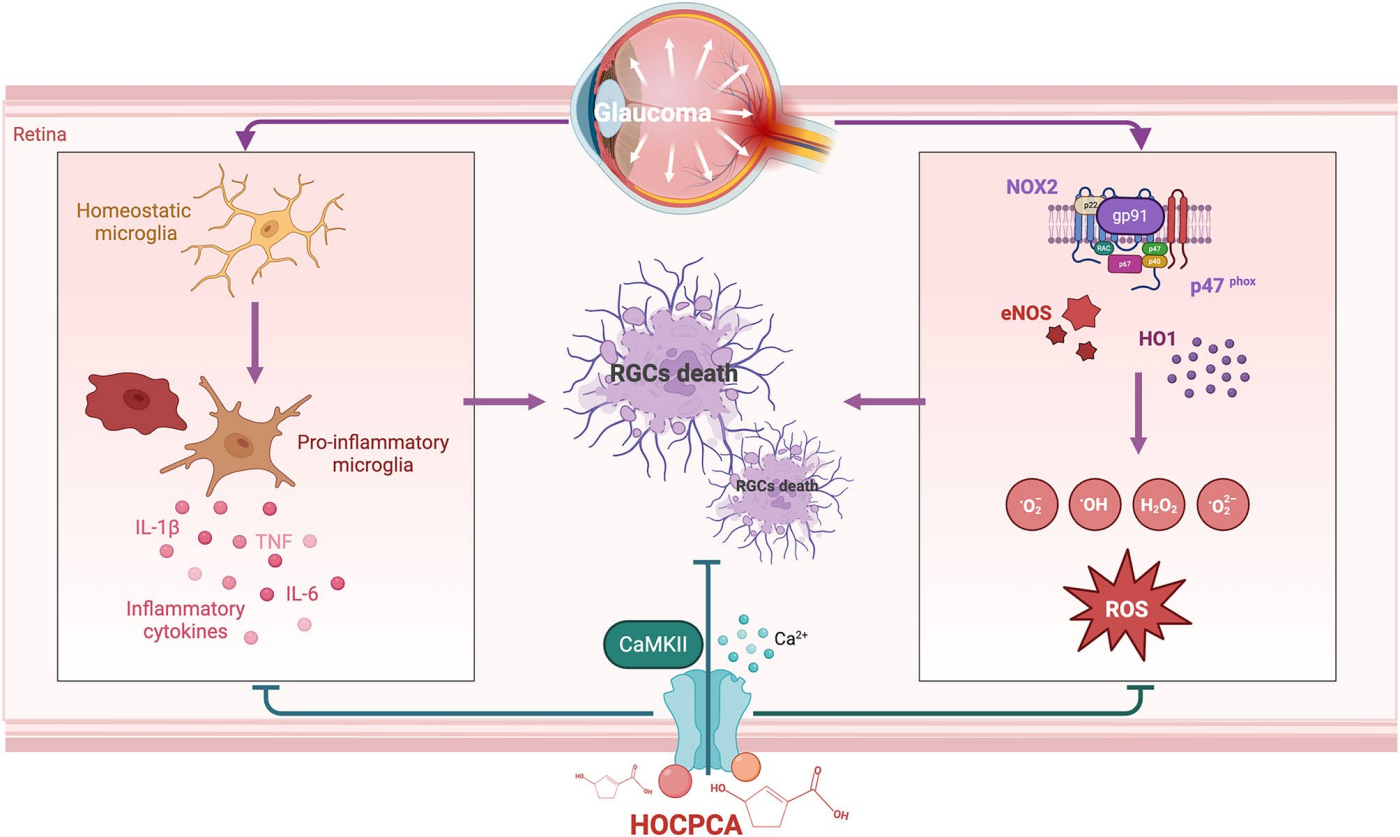
HOCPCA directly protects RGCs by interacting with both CaMKIIα and CaMKIIβ. In addition, it indirectly mitigates glaucomatous RGC loss by reducing microglia-induced neuroinflammatory dysregulation and oxidative stress in the retina under pathologically high pressure. The illustration in Fig. 9 was created using BioRender.com.

In this study, we demonstrated that neuroinflammation, Ca^2+^ signaling, and oxidative stress play critical roles in an *in vivo* model of glaucoma with H-IOP, as revealed by proteomic analysis. CaMKIIα, identified as a key neuronal signaling protein and potential therapeutic target, appears central to the regulation of neuroinflammation, Ca^2+^ signaling, and oxidative stress, based on PPI analysis. HOCPCA, a compound known to bind the CaMKIIα hub domain [21], is likely to exert direct neuroprotection on RGCs through its interaction with both CaMKIIα and CaMKIIβ. In addition, HOCPCA may contribute to an improved retinal environment by modulating microglial activity and reducing oxidative stress, which could provide secondary neuroprotective effects for RGCs. This study is the first to demonstrate the neuroprotective potential of HOCPCA in the retina, highlighting CaMKIIα and β as promising therapeutic targets for glaucoma (Fig. 9).

CaMKII regulates various intracellular processes essential for neuronal homeostasis and plays a critical role in synaptic plasticity [40]. In our study, we first observed the co-localization of CaMKs with RBPMS+ RGCs and identified their intracytoplasmic expression. Aberrant Ca^2+^ activation is linked to excitotoxicity and RGC death following injury, such as optic nerve damage [41, 42]. Elevated IOP, with or without optic nerve fiber loss, has been shown to decrease CaMKIIα protein expression in koniocellular neurons [43]. Excitotoxic injury to RGC somatic cells or optic nerve damage to RGC axons leads to the inactivation of CaMKII and its downstream target, cAMP-responsive element-binding protein, in RGCs [13]. Consistent with our findings, the density of CaMKIIα- and CaMKIIβ-positive cells in the GCL was significantly reduced in retinas subjected to high hydrostatic pressure *in vitro*. A previous study demonstrated the co-localization of CaMKII immunopositive signals with apoptotic RGCs in the retinas of mice two months post-diabetes induction [44]. Loss of CaMKII activity was associated with >80% RGC loss one week after NMDA receptor injection [13]. Blood genetic profiling studies comparing primary open-angle glaucoma patients with normal controls suggest that CaMKII may contribute to its pathogenesis [45]. These studies support our findings and highlight CaMKII's crucial role in neuronal signaling and its potential as a promising therapeutic target for glaucoma.

HOCPCA exhibits submicromolar affinity for the central structural domain of CaMKIIα (*K*_*I*_ 0.13 µmol/L), making it a valuable tool for studying CaMKII neuropharmacology [12]. HOCPCA has been shown to significantly reduce hippocampal neuron death induced by high concentrations of L-glutamate, demonstrating a neuroprotective effect [12]. Our findings show that HOCPCA provides neuroprotection to RGCs under high pressure and oxidative stress, with significant effects at 100 nmol/L. This concentration, close to HOCPCA’s *K*_*I*_ value (130 nmol/L), suggests specific binding to CaMKIIα, minimizing off-target interactions and enhancing neuroprotection. At higher concentrations (1 μmol/L), nonspecific interactions may lead to toxicity, reducing the protective effects. Conversely, lower concentrations (1 nmol/L, 10 nmol/L) may lack sufficient binding affinity, failing to provide adequate neuroprotection.

Reactivating CaMKII protects RGCs in two glaucoma models where RGCs degenerate due to elevated intraocular pressure or genetic deficiency [13]. Our study demonstrates that HOCPCA exerts direct neuroprotection on RGCs through its interaction with both CaMKIIα and CaMKIIβ. This assertion is strongly supported by the evidence that HOCPCA's protective mechanism is based on its specific interaction with the central domain of CaMKIIα [12]. In synaptic activity-induced Ca^2+^ signaling, Ca^2^⁺ influx binds to calmodulin (CaM), activating both CaMKIIα and CaMKIIβ [46]. CaMKIIβ typically forms α/β-CaMKII heteromers with CaMKIIα, facilitating their transport to the postsynaptic density and enhancing synaptic activity through autophosphorylation at T286 and T287 [46, 47]. We observed the co-localization of CaMKIIα and CaMKIIβ in the retinal GCL. Thus, the preservation of CaMKIIβ+ cells is likely to be a secondary effect of the preservation of CaMKIIα+ cells, which in turn could have supported the survival of CaMKIIβ+ cells, rather than a direct action of HOCPCA on CaMKIIβ+ cells. This synergistic mechanism may explain HOCPCA's significant protection of double-positive cells.

HOCPCA treatment has been shown to reduce the mRNA expression of the microglia/macrophage activation marker Iba1 and the cytokine TNF-α in the ischemic cortex three days after thromboembolic stroke and distal MCAO compared to saline [21]. The most likely explanation for reversing the inhibition of hippocampal neuronal activity after distal MCAO is the suppression of inflammatory markers known to impair neuronal function [21, 48]. Activated microglia induce a strong innate immune response, resulting in the production of both pro-inflammatory and anti-inflammatory cytokines [49, 50]. Neuroinflammation plays a critical role in RGC loss in glaucoma [51]. The early activation of microglia occurs following elevated IOP but precedes RGC death [52]. During this phase, microglia adopt an anti-inflammatory phenotype, phagocytosing degenerating or dead RGCs and secreting cytokines such as IL-4, IL-10, IL-13, TGF-β, and arginase-1, which help maintain a non-toxic retinal environment [53–57]. However, in prolonged glaucoma pathology, uncontrolled microglial activation leads to the release of neurotoxic pro-inflammatory cytokines, including TNF-α, IL-6, and IL-1β, exacerbating neurodegeneration [58–61]. The release of IL-1β from necrotic or apoptotic cells mediates the expression of many genes involved in secondary inflammation, leading to an inflammatory cascade and subsequent RGC death [62, 63]. Microglial activation in glaucoma is not only a consequence of initial RGC degeneration [52] but also a contributing factor to secondary RGC death [58]. Our findings show that HOCPCA treatment reduces the pressure-induced activation of microglia in the retinal GCL, leading to decreased expression of pro-inflammatory cytokines. This suggests that HOCPCA's ability to inhibit inflammatory changes could offer a potential neuroprotective mechanism for RGCs. This mechanism is also supported by a previous study in the CNS [21]. The experimental data from primary microglia provide the first direct evidence that HOCPCA has neuroprotective effects mediated by anti-inflammatory mechanisms, specifically through the inhibition of microglial activation and the modulation of the complement system and pro-inflammatory cytokine release, but only at a specific concentration (100 nmol/L).

Inflammation and oxidative stress are closely interconnected in neurodegenerative processes. The pro-inflammatory cytokine TNF-α induces NOX2-dependent ROS production in microglia [64] and has been identified in the aqueous humor of glaucoma patients [65]. Oxidative stress can deplete tetrahydrobiopterin, leading to NOS uncoupling and increased ROS production [66]. This contributes to endothelial dysfunction and a pro-inflammatory state, underlying various retinal diseases [67]. In addition, activated microglia induce ROS production in retinal microvascular cells *via* NOX2 and NOX4 expression [68]. ROS release initiates intracellular signaling cascades, enhancing the expression of pro-inflammatory genes through early activation of NF-κB [69, 70], which amplifies the inflammatory environment, eventually leading to apoptosis, necrosis, or pyroptosis [71]. This bidirectional relationship between inflammation and oxidative stress establishes a detrimental feedback loop that exacerbates cellular damage [72]. HOCPCA may contribute to an anti-inflammatory environment by modulating microglial activity and reducing oxidative stress; however, whether these effects play a primary or secondary role in its neuroprotective mechanism remains to be determined.

Although previous studies have linked CaMKII to inflammatory molecules like TNF-α and NF-κB [73], as well as T cell receptors [74], our data suggest that CaMKIIα expression in microglia is limited, as confirmed by immunofluorescence and Western blot analysis (see Supplementary Data). Given this, it is unlikely that HOCPCA exerts its neuroprotective effects *via* direct modulation of CaMKII in microglia. HOCPCA likely exerts neuroprotection in RGCs primarily through its direct interaction with CaMKIIα and CaMKIIβ, thereby preserving Ca^2^⁺ homeostasis and neuronal integrity under pathological conditions.

In addition, CaMKII plays a role in oxidative stress signaling [75] regulating cytosolic Ca^2^⁺, gene transcription, and cell survival [76–78]. CaMKII is involved in transient bradykinin-driven eNOS activation *in vitro* but does not regulate NO production [79]. The endoplasmic reticulum (ER) stress signaling pathway involving Ca^2^⁺ and CaMKII induces NADPH oxidase and oxidative stress, both essential for ER stress-induced apoptosis [80]. In ischemic stroke, CaMKIIα inhibits ROS production *in vivo* and increases the GSH/GSSG ratio [81]. Oxidized CaMKII (ox-CaMKII) links inflammatory responses and physiological ROS signaling, promoting inflammatory gene expression [82]. While GHB has been shown to prevent oxidative damage [83, 84], HOCPCA’s role in oxidative stress modulation remains unclear. Whether HOCPCA’s ROS reduction in elevated hydrostatic pressure retinas is a direct or inflammation-mediated effect requires further investigation.

Future studies are needed to explore alternative pathways through which HOCPCA exerts its anti-inflammatory effects. Exploring its effects on ox-CaMKII levels and other oxidative stress regulatory pathways will be essential for understanding its neuroprotective effects. Ultimately, clarifying these mechanisms will be crucial for establishing HOCPCA as a therapeutic candidate for glaucoma and other neurodegenerative diseases.

## Conclusion

In conclusion, our study underscores that HOCPCA provides neuroprotection to RGCs primarily through its direct interaction with CaMKIIα and CaMKIIβ, preserving neuronal homeostasis under conditions of elevated intraocular pressure and oxidative stress. In addition, HOCPCA may contribute to an anti-inflammatory and antioxidative environment in the retina, potentially mitigating secondary neurodegeneration. These findings suggest that HOCPCA can protect RGCs and holds promise as a potential therapeutic agent for glaucoma. However, further research is necessary to fully elucidate its underlying mechanisms and optimize its therapeutic efficacy.

## Supplementary Information

Below is the link to the electronic supplementary material.

Supplementary file1 (PDF 145 KB)

## Data Availability

All data generated or analyzed during this study are included in this published article [and its supplementary information files].
