# Supplementary material for: HOCPCA Exerts Neuroprotection on Retinal Ganglion Cells by Binding to CaMKIIα and Modulating Oxidative Stress and Neuroinflammation in Experimental Glaucoma
Source: Neurosci Bull. 2025 Jun 2;41(8):1329–46. doi: 10.1007/s12264-025-01417-0 (PMC12314278; doi:10.1007/s12264-025-01417-0)
Supplement: Supplementary file 1 — Supplementary file1 (PDF 145 KB) [file 12264_2025_1417_MOESM1_ESM.pdf]

Supplementary Data

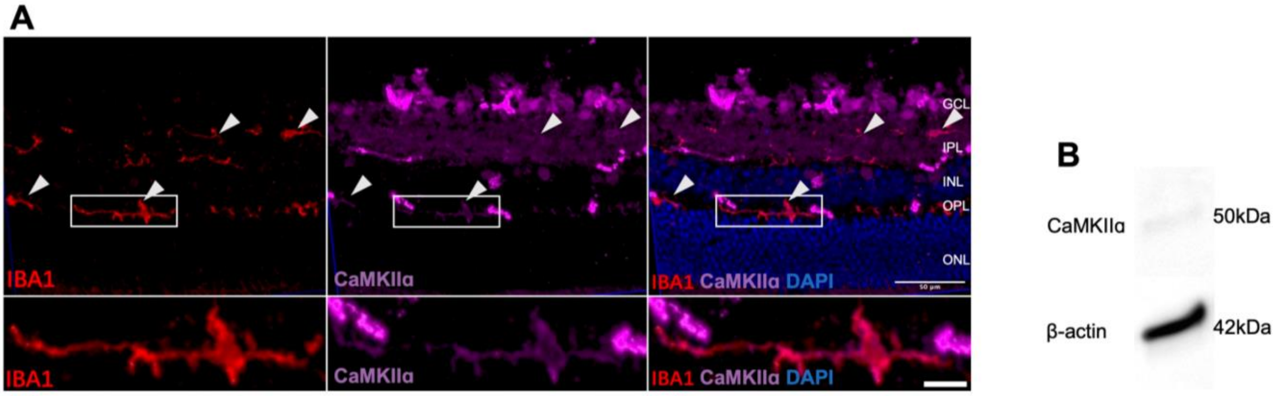

Supplementary Figure:

(A) Colocalization analysis of IBA1-positive microglia and CaMKIIα in the retina. Immunofluorescence staining reveals the colocalization of IBA1 (red) and CaMKIIα (purple) in frozen retinal sections under physiological conditions. DAPI (blue) was used for nuclear staining to visualize the layered structure of the retina. The magnified view of the white rectangular region further highlights the colocalization of $IBA1^+$ microglia with CaMKIIα. Scale bars: 50 μm (main image); 10 μm (magnified view).

(B) Western blot analysis of target protein expression in primary microglial cell lysates.
